# Expanding the immunotherapy universe in extensive-stage small cell lung cancer: from chemoimmunotherapy backbone to next-wave combinations

**DOI:** 10.3389/fimmu.2025.1693401

**Published:** 2025-12-03

**Authors:** Xiang Chi, Yan Dong, Lide Zhu, Di Su, Haolin Wu

**Affiliations:** 1 Department of Oncology and Hematology, The Affiliated Hospital to Changchun University of Chinese Medicine, Changchun, Jilin, China; 2 Department of Outpatient Services, The Affiliated Hospital to Changchun University of Chinese Medicine, Changchun, Jilin, China; 3 Department of Internal Medicine, The Affiliated Hospital to Changchun University of Chinese Medicine, Changchun, Jilin, China; 4 Department of Rehabilitation Medicine, Tong Hua City Hospital of Chinese Medicine, Tonghua, Jilin, China

**Keywords:** extensive-stage small cell lung cancer, immunotherapy combinations, anti-angiogenic agents, radiotherapy, targeted therapy, emerging research

## Abstract

Small-cell lung cancer (SCLC) is a highly malignant neuroendocrine tumor characterized by rapid proliferation and dismal prognosis. Platinum-based chemotherapy combined with immune checkpoint inhibitors (ICIs) is now the first-line treatment for extensive-stage disease (ES-SCLC), extending the overall survival (OS) period of these patients by 2–5 months, yet durable remissions remain the privilege of fewer than 20% of patients. Despite intensive investigation, this incremental benefit appears to have plateaued, prompting exploration of alternative combination strategies to unleash deeper and more durable antitumor synergy. Recent phase II/III trials integrating anti-angiogenic agents into the chemo-immunotherapy have reported unprecedented OS gains of up to 7 months, redefining therapeutic expectations. Concurrently, chemoradiation with ICIs triplet regimens have demonstrated encouraging antitumor activity in ES-SCLC, while rational combinations of small-molecule targeted drugs (DLL3 inhibitors, PARP inhibitors) combined with ICIs or epigenetic modifiers with ICIs are yielding early signals of efficacy. Nevertheless, primary resistance, absence of robust predictive biomarkers, and cumulative toxicity continue to curtail clinical impact. This Review provides a comprehensive, evidence-based map of the evolving ES-SCLC immunotherapy combination landscape. We critically dissect competing therapeutic paradigms, juxtapose corroborative and contradictory data, and distill actionable insights for future trial design, biomarker development, and regulatory strategy.

## 1 Introduction

Lung cancer has the highest incidence and mortality rates among all types of malignant tumors (1, 2). In 2022, it was responsible for approximately 2.5 million new cases worldwide (accounting for 12.4% of all new cancer cases), and led to 1.8 million deaths, with a mortality rate of 18.7% (2). Lung cancer is mainly divided into two types: non-small cell lung cancer (NSCLC) and small cell lung cancer (SCLC) (3). SCLC accounts for approximately 15% of all lung cancers and is characterized by a high proliferative rate, strong predilection for early metastasis and poor prognosis (4, 5). Approximately 250,000 SCLC patients are diagnosed each year globally, of which approximately 200,000 succumb to this disease. According to the Veterans Administration Lung Study Group (VALG) staging system, SCLC is classified into limited-stage SCLC (LS-SCLC) and extensive-stage SCLC (ES-SCLC) (4). Approximately 70% of patients are diagnosed with ES-SCLC on initial examination. The 5-year survival rate for LS-SCLC is only 10%-15%, while for ES-SCLC, it is even lower at 1%-2% (6–8).

ES-SCLC exhibit greater tumor heterogeneity (9). For decades, platinum-based drugs (cisplatin or carboplatin) combined with etoposide in a two-drug chemotherapy has been the standard first-line treatment for ES-SCLC (10). While this treatment demonstrates remarkable short-term anti-tumor effects, the objective response rate (ORR) reached up to 70%, but resistance develops rapidly (11, 12). Furthermore, the prognosis for ES-SCLC remains poor, with a median survival typically ranging from 8 to 10 months (11, 13). The rapid development of immunotherapy has significantly transformed the treatment for ES-SCLC, bringing substantial survival benefits to patients (14). Multiple randomized phase III studies have shown that incorporating immune checkpoint inhibitors (ICIs) into first-line chemotherapy for newly diagnosed ES-SCLC patients results in statistically significant benefits (15–20). This approach has demonstrated the efficacy and safety of ICIs in tumor control and has enhanced survival outcomes, extending the survival period by 2 to 6 months.

Chemoimmunotherapy is currently the first-line standard treatment for ES-SCLC. However, fewer than 20% of those patients achieve long-term survival (21). The tumor microenvironment and the reduction in immunogenicity are two key mechanisms of immunotherapy resistance for PD-1/PD-L1 blockade, which lead to limited responses (22). Therefore, optimizing treatment regimens to further prolong survival in ES-SCLC patients remains a critical challenge in clinical practice. The combinations that enhance the efficacy of ICIs and expand their indications has stood out (23, 24). Three competing paradigms define ES-SCLC combination therapy: (i) Immune-Maintenance, asserting that chemo-immunotherapy followed by ICI maintenance reaches an efficacy plateau (25); (ii) Early-Radiotherapy, proposing cycle-2 thoracic irradiation to ignite an immune-cold microenvironment (26); and (iii) Anti-Angiogenesis, advocating VEGF inhibition to remodel tumor vessels without excess myelotoxicity (27). In addition, emerging data demonstrate that DLL3-directed bispecific T-cell engagers (BiTEs), PARP inhibitors combined with ICIs also show activity (28, 29). By combining ICIs with other therapeutic approaches, a synergistic effect can be achieved, enhancing the anti-tumor immune response, overcoming resistance mechanisms, and improving treatment efficacy. This review elaborates on the mechanisms, clinical applications, and challenges faced by immunotherapy combinations.

## 2 Molecular characteristics and subtypes of SCLC

The molecular mechanism underlying the pathogenesis of SCLC remain incompletely understood. Inactivating mutations in *TP53* and *RB1*, which occur in nearly 90% of SCLC cases, foster a genomic landscape marked by unchecked proliferation and a deficient DNA damage response (30). Beyond these core drivers, whole-exome sequencing has identified additional recurrent alterations, including *MYC* family amplifications (in 20–30% of cases), NOTCH pathway mutations (~25%), and disruptions in chromatin-modifying genes such as *KMT2C/D* and *CREBBP* (31, 32). While these genomic alterations define the initiating events in ES-SCLC, their functional consequences do not fully manifest at the DNA level. Instead, they converge to drive profound transcriptional heterogeneity, which is captured by the following molecular subtypes. Early classification schemes divided SCLC into two molecular subtypes according to the expression levels of the transcription factors achaete-scute homologue 1 (ASCL1; also known as ASH1) and neurogenic differentiation factor 1 (NeuroD1), establishing a foundational dichotomy for future research (33). Rudin et al. subsequently established the canonical four-subtype system (SCLC-A, -N, -P, -Y) (34). This framework introduced a clear biological dichotomy between neuroendocrine (NE)-high (comprising SCLC-A, SCLC-N) and NE-low (comprising SCLC-P, SCLC-Y) subgroups, each driven by its respective transcription factor (ASCL1, NEUROD1, POU class 2 homeobox 3 (POU2F3) and yes-associated protein 1 (YAP1), respectively). Gay et al. refined this paradigm by identifying an inflamed subtype, SCLC-I, which is characterized by the concurrent loss of ASCL1, NEUROD1, and POU2F3 expression and a prominent immune signature (35). Further expanding this landscape, recent profiling efforts have proposed additional categories, including an SCLC-AN subtype with co-expression of ASCL1 and NEUROD1, and a quadruple-negative (SCLC-QN) subtype lacking all four canonical biomarkers (36). Most recently, Liu et al. integrated multi-omics data through non-negative matrix factorization (NMF) to validate these subtypes and reveal additional biological dimensions (32). The nmf1 subtype corresponds to SCLC-A/N with high neuroendocrine scores and frequent ASCL1/NEUROD1 co-expression; nmf3 displays the highest epithelial-mesenchymal transition (EMT) score, correlating with metastasis and chemoresistance; and nmf4 (SCLC-P) shows exclusive POU2F3 expression and *MYC*-driven metabolic reprogramming, particularly purine synthesis dependency. Research into molecular classification has elucidated the unique molecular signatures of SCLC subtypes and their heterogeneous responses to chemoimmunotherapy, targeted-agents, underscoring the rationale for crafting precision therapeutic strategies. This review will focus on the section “ICIs + targeted therapy” for ES-SCLC and elaborate on how molecular subtypes can provide a basis for treatment selection and predict treatment sensitivity.

## 3 Targets and mechanisms of immune checkpoints

**Figure 1:**
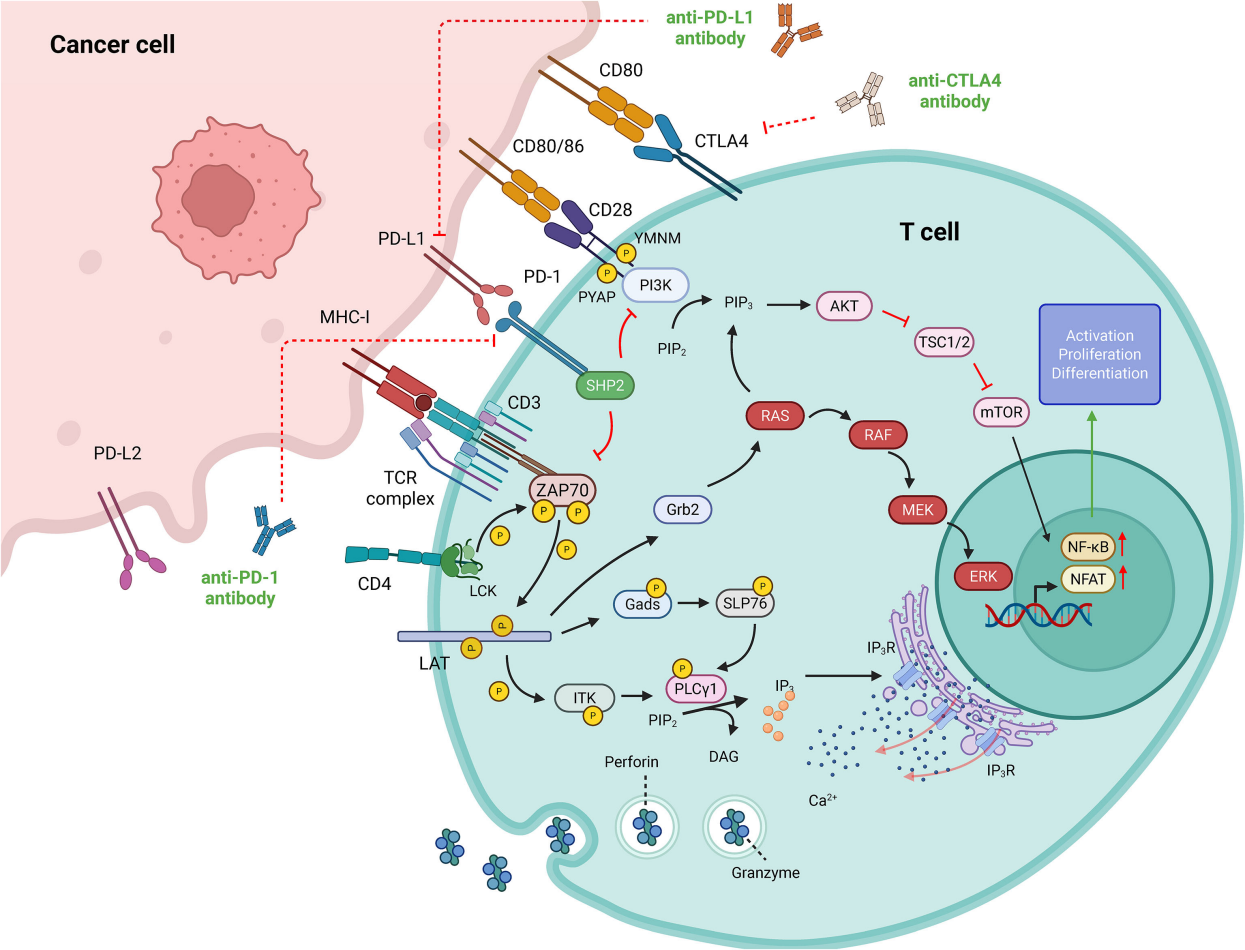
Molecular handshake at the immune synapse: how CTLA-4 and PD-1 silence cytotoxic T cells (By adobe illustrator). The intricate interactions between T cells and tumor cells involve a dynamic interplay that is modulated by immune checkpoint inhibitors. These inhibitors enhance the anti-tumor capabilities of T cells by blocking the PD-1/PD-L1 and CTLA-4 signaling pathways, effectively relieving the inhibitory signals that dampen T cell activity. This mechanism forms the theoretical foundation for cancer immunotherapy, which aims to bolster the body’s own immune system to fight against tumors.

To fully grasp the mechanisms underlying ICIs, it is essential to appreciate the diverse immune functions they modulate. In the context of SCLC immunotherapy, the two most extensively studied immune checkpoint receptors are cytotoxic T lymphocyte-associated antigen 4 (CTLA-4, also called CD152) and programmed cell death protein 1 (PD-1, also called CD279) (37). Both are inhibitory receptors that regulate immune responses at distinct stages via unique mechanisms. This review focuses on the CTLA-4 and PD-1 pathways. Additionally, numerous potential targets and novel ICIs are currently being actively explored (**Figure 1**).

### 3.1 CTLA-4

CTLA-4 is exclusively expressed on T cells and plays a pivotal role during the initial activation phase of these cells. In resting T cells, CTLA-4 predominantly exists as an intracellular protein. However, upon T cell receptor (TCR) engagement and receipt of co-stimulatory signals mediated by CD28, CTLA-4 translocates to the cell surface (38, 39). CTLA-4 shares structural homology with CD28 but binds CD80 (B7-1)/CD86 (B7-2) with >10-fold higher affinity (40, 41). This high-avidity ligation recruits PKC-η, disassembles the PIX–GIT2–PAK2 complex and thereby acutely shuts down TCR-proximal signaling, halting T-cell proliferation and activation (42, 43). Moreover, CTLA-4 sequesters CD28 from binding to CD80/CD86 and actively removes these ligands from the surface of antigen-presenting cells (APCs), exerting “signal-independent” T cell inhibition (44, 45). In the tumor microenvironment, the high expression of CTLA-4 facilitates the evasion of tumor cells from the immune system’s attack and maintains an immunosuppressive state.

### 3.2 PD-1/PD-L1

PD-1, an immune checkpoint molecule primarily expressed on the surface of immune cells such as T cells, B cells, and natural killer (NK) cells, transmits inhibitory signals by binding to programmed death ligand 1(PD-L1) and programmed death ligand 2 (PD-L2) (46, 47). PD-1 mainly inhibits the cytotoxic function of T cells during their effector phase. The intracellular tail of PD-1 contains two tyrosine-based signaling motifs: immunoreceptor tyrosine-based inhibitory motif (ITIM) and immunoreceptor tyrosine-based switch motif (ITSM) (48). When PD-1 binds to PD-L1 or PD-L2, ITIM and ITSM become phosphorylated, recruiting and activating src homology 2 domain-containing tyrosine phosphatase 2 (SHP-2) (49). Activated SHP-2 dephosphorylates a series of signaling molecules downstream of TCR and CD28, including zeta-chain-associated protein kinase 70 (ZAP70), src-like adapter protein of 76 kda (SLP-76), protein kinase C θ (PKC-θ), phosphoinositide-3-kinase (PI3K), and the ras signaling pathway, thereby inhibiting T cell activation and function (50, 51). Tumor cells can evade immune attacks by overexpressing PD-L1, which binds to PD-1 on T cells (52, 53). PD-L1-induced PD-1 oligomerization requires the phosphorylation of the PD-1 intracellular tail. The ITSM of PD-1 can bind to the N-terminal src homology 2 domain (N-SH2) and C-terminal src homology 2 domain (C-SH2) domains of SHP-2, inducing PD-1 dimerization, enhancing SHP-2 protein tyrosine phosphatase activity, and further inhibiting TCR or CD28 signaling (54). This mechanism allows tumor cells to evade immune system attacks.

### 3.3 Immune checkpoint inhibitors

CTLA-4 inhibitors and PD-1/PD-L1 inhibitors are currently the two most extensively studied ICIs in the treatment of SCLC. The US Food and Drug Administration (FDA) approved ipilimumab, an antibody targeting CTLA-4, as the first immune checkpoint inhibitor for the treatment of advanced metastatic melanoma in 2011 (55, 56). The continuous development of immunotherapy in the field of oncology has led researchers to focus on SCLC. The traditional treatment methods for SCLC include chemotherapy and radiotherapy, but the long-term survival rate remains relatively low. Therefore, exploring new treatment approaches is of great significance.

#### 3.3.1 CTLA-4 inhibitors

CTLA-4 inhibitors achieve this by blocking the binding of CTLA-4 to CD80/CD86, restoring the co-stimulatory signal of CD28, enhancing the activation and function of T cells, and thereby increasing the immune system’s ability to attack tumors. CTLA-4 inhibitors, including ipilimumab and tremelimumab, have been investigated in SCLC but failed to demonstrate clinical benefit (57, 58). Recently, the combination of CTLA-4 inhibitors and PD-1/PD-L1 inhibitors has been used to treat ES-SCLC, and has shown certain anti-tumor activity (59–63). Blockade of the PD-1/PD-L1 axis mainly eliminates T cell exhaustion within the tumor microenvironment, while blockade of CTLA-4 promotes the efficient activation and clonal expansion of naive T cells in peripheral lymph nodes. The preclinical data from 2010 indicated that the combination of anti-CTLA-4 and anti-PD-1 therapies expanded infiltrating T cells and reduced regulatory T and myeloid cells, having a higher response rate than using either drug alone (64). Recently, CheckMate-032 reported an ORR of 21.9% with ipilimumab plus nivolumab in SCLC, but grade≥3 immune-related adverse events (iRAEs) occurred in 37.5% of patients (65, 66). CheckMate-451 subsequently failed to demonstrate OS benefit (HR = 0.92, P = 0.37) and showed even higher toxicity with grade≥3 iRAEs in 52.2% of patients (59). Amid the modest durability and substantial iRAEs that have limited the clinical utility of conventional CTLA-4 inhibitors with PD-1/PD-L1 inhibitors combinations in SCLC, the advent of PD-1/CTLA-4 bispecific antibodies—exemplified by cadonilimab—offers a mechanistically refined strategy that maintains potent antitumor activity. Cadonilimab (AK104) is a tetravalent bispecific IgG-single-chain Fv fragment (ScFv) antibody that simultaneously targets PD-1 and CTLA-4 (67). Approved in China in June 2022 for relapsed/metastatic cervical cancer after platinum failure, the agent has also shown broad activity in advanced solid tumors (68). A multicenter phase II trial (NCT05308784) is evaluating cadonilimab ± second-line treatment in ES-SCLC; detailed results have not yet been disclosed (69). There are no clinical trials specifically targeting SCLC with PD-1/CTLA-4 bispecific antibodies. Recently approved by Qilu pharmaceutical, the co-formulated anti-PD-1/CTLA-4 pair—Iparomlimab and tuvonralimab—has entered the armamentarium against advanced solid tumors, and its built-in dual-checkpoint blockade positions the regimen as an immediately exploitable backbone for SCLC combinations, potentially redefining second-line or maintenance strategies when layered onto chemotherapy, radioligand or cellular therapies (70). Most ongoing clinical trials mainly focus on other solid tumors (68, 71). However, the potential application of these agents in SCLC warrants further exploration. Future research will focus on the combined application of CTLA-4 inhibitors with other treatment methods, aiming to enhance the therapeutic effect and overcome the problem of drug resistance. CTLA-4 inhibitors and PD-1/CTLA-4 bispecific antibodies currently undergoing clinical trials are listed in following **Table 1**.

**Table 1:** The CTLA-4 inhibitors and PD-1/CTLA-4 bispecific antibodies currently in the clinical trial stage for solid tumors or lung cancer.

Clinical trials.gov ID	Drug	Clinical Trial Registration URL	Phase	Cancer stage	Status
CTLA-4 inhibitors					
NCT04501276	ADG116	https://clinicaltrials.gov/ct2/show/NCT04501276	I	Advanced solid tumors	Active, not recruiting
NCT04699929	YH001	https://clinicaltrials.gov/ct2/show/NCT04699929	I	Advanced solid tumors	Completed
NCT04336241	RP2	https://clinicaltrials.gov/ct2/show/NCT04336241	I	Advanced solid tumors	Recruiting
NCT03860272	Botensilimab	https://clinicaltrials.gov/ct2/show/NCT03860272	I	Advanced solid tumors	Active, not recruiting
NCT03523819	CS1002	https://clinicaltrials.gov/ct2/show/NCT03523819	I	Advanced solid tumors	Completed
NCT04126590	KN044	https://clinicaltrials.gov/ct2/show/NCT04126590	I	Advanced solid tumors	Recruiting
PD-1/CTLA-4 bispecific antibodies					
NCT05505825	AK104	https://clinicaltrials.gov/ct2/show/NCT05505825	I/II	ES-SCLC	Completed
NCT05901584	AK104	https://clinicaltrials.gov/ct2/show/NCT05901584	I/II	ES-SCLC	Unknown
NCT04646330	AK104	https://clinicaltrials.gov/ct2/show/NCT04646330	I/II	NSCLC	Active, not recruiting
NCT04544644	AK104	https://clinicaltrials.gov/ct2/show/NCT04544644	II	NSCLC	Unknown
NCT07091305	QL1706	https://clinicaltrials.gov/ct2/show/NCT07091305	II	LS-SCLC	Active, not recruiting
NCT03819465	MEDI5752	https://clinicaltrials.gov/ct2/show/NCT03819465	I	NSCLC	Active, not recruiting
NCT03530397	MEDI5752	https://clinicaltrials.gov/ct2/show/NCT03530397	I	Advanced solid tumors	Active, not recruiting
NCT03517488	XmAb20717	https://clinicaltrials.gov/ct2/show/NCT03517488	I	Advanced solid tumors	Completed
NCT03761017	MGD019	https://clinicaltrials.gov/ct2/show/NCT03761017	I	Advanced solid tumors	Completed
NCT04054531	KN046	https://clinicaltrials.gov/ct2/show/NCT04054531	II	NSCLC	Unknown
NCT04474119	KN046	https://clinicaltrials.gov/ct2/show/NCT04474119	III	Advanced squamous NSCLC	Unknown

#### 3.3.2 PD-1/PD-L1 inhibitors

PD-1/PD-L1 inhibitors liberate antitumor immunity by interrupting the inhibitory axis between T-cell PD-1 and PD-L1/PD-L2 expressed on SCLC tumor cells and on tumor-infiltrating macrophages or dendritic cells (72). Antibodies such as nivolumab/pembrolizumab/serplulimab/tislelizumab/(anti-PD-1) or atezolizumab/durvalumab (anti-PD-L1) prevent PD-1–PD-L1/PD-L2 and PD-L1–B7-1 (CD80) engagements, thereby relieving SHP-2–mediated suppression of TCR signaling (15–17, 73–75). This restores CD8^+^ T-cell proliferation, cytotoxic granule release, and IFN-γ secretion, while simultaneously enhancing dendritic-cell antigen presentation. Although SCLC cells display scant PD-L1 (~5%), 18.5–56.3% of intratumoral immune cells express PD-L1, implicating this stromal ligand as a key mediator of immune evasion and a critical target for PD-1/PD-L1-directed therapy. Strategies combining PD-1/PD-L1 inhibitors combined with chemotherapy have demonstrated success and have reshaped the treatment landscape for ES-SCLC (15–17, 73–75). Currently, first-line treatment for ES-SCLC is the combination of ICIs with platinum-based chemotherapy, followed by maintenance therapy with ICIs (15, 16). The following text will provide a detailed account of the anti-tumor effects of PD-1/PD-L1 inhibitors in ES-SCLC patients and elaborate on more treatment regimens combining immune checkpoints for SCLC.

## 4 Immune checkpoints inhibitors combination therapy

**Figure 2:**
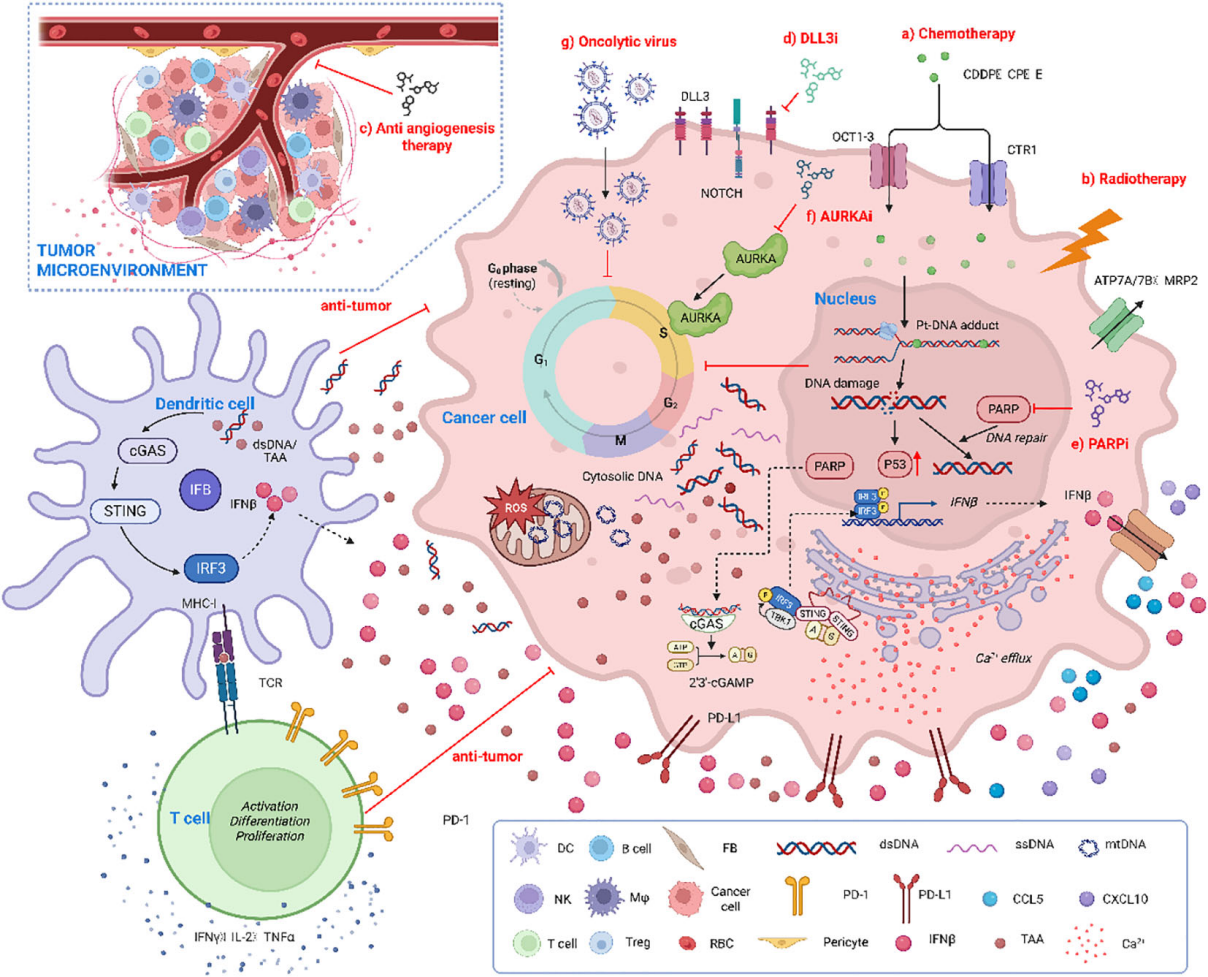
Synergistic anti-tumor efficacy of multimodal immunotherapy combinations in ES-SCLC (By adobe illustrator). Within the tumor microenvironment (TME), a multitude of therapeutic strategies synergistically augment anti-tumor T cell responses through the induction of DNA damage, activation of innate immune sensing pathways, and alleviation of immune suppression. These strategies include: **(a)** Chemotherapy (CDDP/cisplatin, CP/carboplatin, E/etoposide) leads to the formation of Pt-DNA adducts, resulting in DNA double-strand breaks; **(b)** Radiotherapy directly generates DNA damage and produces cytoplasmic double-stranded DNA (dsDNA); **(c)** Anti-angiogenic therapy inhibits tumor blood vessels, thereby enhancing T cell infiltration into the tumor; **(d)** DLL3-targeted therapy (DLL3i) selectively eliminates DLL3-expressing tumor cells; **(e)** PARP inhibitor (PARPi) inhibits PARP-mediated DNA repair processes, exacerbating DNA damage; **(f)** AURKA inhibitor (AURKAi) inhibits Aurora Kinase A, inducing mitotic catastrophe in tumor cells; **(g)** Oncolytic viruses replicate within tumor cells, leading to tumor lysis and the release of dsDNA and tumor-associated antigens (TAAs). Cell damage prompts the release of dsDNA, ssDNA, and mtDNA into the cytoplasm. The cGAS-STING pathway detects this DNA, leading to IFNβ production, which enhances dendritic cell (DC) antigen presentation and T cell activation. DCs presenting TAAs to T cells, combined with IFNβ effects, stimulate T cell activation, proliferation, and differentiation. Activated T cells secrete cytokines like IFN-γ, IL-2, and TNF-α, further modulating the immune response and recruiting more CD8^+^ T cells via chemokines such as CCL5 and CXCL10. Additionally, B cells, RBCs, and pericytes contribute to TME regulation. This integrated strategy strengthens the body’s immune system against tumors, providing a foundation for cancer immunotherapy.

ICIs reinvigorate intratumoral cytotoxic T cells; combination therapy further exposes tumor antigens and directs T-cell–mediated killing, thereby amplifying antitumor efficacy (72). The highly proliferative characteristic of SCLC is more susceptible to DNA damage and cell apoptosis induced by chemotherapy or radiotherapy (76). Although chemotherapy and radiotherapy both induce tumor-cell death and antigen release to potentiate immunotherapy, their underlying anti-SCLC mechanisms differ and will be delineated below. Encouragingly, ongoing trials integrating anti-angiogenic agents or chemoradiation with ICIs, together with later-line strategies such as DLL3-directed BiTEs, PARP inhibitors, and lurbinectedin plus ICIs, have all demonstrated measurable antitumor activity. Nevertheless, primary resistance, a paucity of predictive biomarkers, and cumulative toxicity continue to curtail clinical benefit. The following sections systematically chart the current immune-combination landscape, dissect the competing paradigms, present the supporting and dissenting evidence for each, and outline future directions and trial-design recommendations (**Figure 2**).

### 4.1 ICIs + chemotherapy

#### 4.1.1 First-line chemoimmunotherapy

Etoposide plus platinum (e.g., cisplatin/carboplatin) remains the standard first-line treatment for ES-SCLC (77). Platinum agents coordinate to the N7 positions of purine bases in DNA, forming bifunctional cisplatin–purine adducts that distort the double helix, stall replication forks, and trigger DNA-damage signaling cascades, culminating in cell-cycle arrest and apoptosis (78, 79). Etoposide, topoisomerase II inhibitor, intercalates into the enzyme–DNA cleavage complex and physically blocks the re-ligation step, thereby stabilizing the normally transient complex formed between topoisomerase II and the 5′-cleaved ends of DNA. This trapping prevents the resealing of DNA double-strand breaks (DSBs), leading to the accumulation of persistent, protein-linked DSBs that overwhelm cellular repair capacity and ultimately trigger apoptosis (80). Second-line topotecan or irinotecan traps topoisomerase I–DNA cleavage complexes; stalled replication forks convert these single-strand nicks into double-strand breaks, triggering apoptosis in SCLC cells (81–83). Chemotherapy rapidly debulks the tumor, liberating abundant tumor-associated antigens (TAAs) while transiently rewiring the microenvironment. The surviving cancer cells up-regulate PD-L1, sensitizing them to PD-1/PD-L1 blockade. Concurrent or sequential administration of PD-1/PD-L1 inhibitors releases the brakes on pre-existing and neo-expanded CD8^+^ T cells, converting the antigen surge into durable cytotoxic activity. Continued single-agent ICI maintenance then sustains T-cell memory, extending survival.

The FDA approved the combination of PD-L1 inhibitor atezolizumab with chemotherapy for the first-line treatment of ES-SCLC patients in 2019 (15). This marked a new milestone in the treatment of SCLC and brought new hope for the treatment of ES-SCLC. Horn et al. reported that atezolizumab in combination with carboplatin and etoposide prolonged median overall survival (OS) by 2.0 months compared with chemotherapy alone (12.3 months vs 10.3 months), demonstrating a significant benefit (hazard ratio (HR) 0.70; 95% confidence interval (95% CI) 0.54–0.91; P = 0.007) in the IMpower133 trial (15). The updated clinical data further confirmed the application value of atezolizumab in the maintenance treatment of ES-SCLC patients (73, 74). Moreover, Paz-Ares et al. reported that first-line treatment with durvalumab plus platinum and etoposide for ES-SCLC prolonged the median OS by 2.7 months (13.0 months vs 10.3 months; HR = 0.73; 95% CI 0.59 - 0.91; P = 0.0047) in the CASPIAN phase III study (18). However, the improvement in OS achieved by the approved PD-L1 inhibitors was moderate, indicating that the clinical need for more effective treatments among ES-SCLC patients has not been met. Cheng et al. demonstrated that first-line serplulimab combined with etoposide and platinum significantly prolonged median OS by 4.5 months compared with chemotherapy alone (15.4 vs 10.9 months; HR = 0.63; 95% CI 0.49–0.82; P < 0.001) in ES-SCLC patients in the phase III ASTRUM-005 trial (18). Updated findings confirm that serplulimab continues to confer durable clinical benefit over placebo in ES-SCLC (84). Exploratory analyses further indicate that a 15-protein signature and alterations in RB1 or Notch pathway genes may serve as predictive biomarkers for therapeutic response. Serplulimab is the first PD-1 inhibitor to yield a statistically significant and clinically meaningful OS benefit in the first-line treatment of ES-SCLC, establishing a new standard of care for this population. In addition, Wang et al. reported in the CAPSTONE-1 Phase III study: adebrelimab (PD-L1 inhibitors) combined with carboplatin and etoposide as first-line treatment for ES-SCLC, extended the median OS from 10.8 months (chemotherapy group) to 15.3 months; HR = 0.72 (95% CI 0.58 - 0.90; P = 0.004) (17). Recently, tislelizumab and toripalimab both conferred modest survival gains in first-line ES-SCLC, yet the absolute OS extension was only about 2 months (19, 20).

Now, IMpower133 and CASPIAN are two major Phase III trials that have established global standards (FDA/European Medicines Agency (EMA)), and they respectively supported the approval of atezolizumab and durvalumab in ES-SCLC (14–16). The four studies, ASTRUM-005, CAPSTONE-1, RATIONALE-312, and EXTENTORCH, all originated from multi-center Phase III trials led by China and have been approved by the National Medical Products Administration (NMPA) and incorporated into the Chinese Society of Clinical Oncology (CSCO) guidelines (17–20). With researchers delving deeper into PD-1/PD-L1 inhibitors, an increasing number of ICIs are being applied in ES-SCLC patients, significantly improving their survival rates while demonstrating good safety. However, the widespread adoption of immune-checkpoint combinations has been accompanied by a rising incidence of iRAEs—including life-threatening myocarditis and pneumonitis—necessitating vigilant monitoring and individualized management by clinicians (85, 86). Therefore, continued investigation is warranted to refine patient selection and to develop combination strategies that can translate the biological promise of PD-1/PD-L1 blockade into more durable clinical benefit.

#### 4.1.2 Second-line chemotherapy

Second-line topotecan or irinotecan traps topoisomerase I–DNA cleavage complexes; stalled replication forks convert these single-strand nicks into double-strand breaks, triggering apoptosis in SCLC cells (81–83). However, treatment options after progression on first-line chemoimmunotherapy for ES−SCLC remain limited, with no conclusive evidence supporting second-line combinations of chemotherapy and ICIs. Lurbinectedin is a recently FDA-approved second-line treatment for ES-SCLC after platinum-based chemotherapy based on the Phase II basket trial (Study B-005) (87). Pre-clinical and early clinical findings indicate that lurbinectedin acts as an immunostimulatory DNA-damage agent in SCLC (88, 89). By engaging the STING pathway, the drug promotes type-I interferon secretion, up-regulates MHC-I/II, and re-programs the tumor microenvironment toward a CD8^+^ T-cell- and M1 macrophage-dominant phenotype while suppressing M2 macrophages (90, 91). These changes markedly enhance the activity of PD-L1 blockade in both first-line and maintenance settings, and the benefit is lost upon STING or CD8 depletion. Consistent with mouse models, patient biopsies show increased MHC-I/II and CD8 after lurbinectedin exposure, supporting its potential to synergize with immunotherapy in SCLC (90, 91). Critically, the phase III IMforte trial demonstrated that lurbinectedin plus atezolizumab as maintenance therapy (after first-line induction, not at second-line progression) significantly improved progression-free survival (5.4 vs. 2.1 months; HR 0.54, p < 0.0001) and overall survival (13.2 vs. 10.6 months; HR 0.73, p = 0.0174) compared with atezolizumab alone (92, 93). Although well tolerated, this combination remains investigational for second-line use after disease progression, where evidence for chemo-immunotherapy remains lacking.

### 4.2 ICIs + radiotherapy

Ionizing radiation (IR) generates reactive oxygen species (ROS) and directly causes DNA DSBs. The broken DNA fragments leak from the nucleus into the cytoplasm and are recognized by cyclic GMP–AMP synthase (cGAS) (94). cGAS catalyzes 2′3′-cGAMP, which binds with high affinity to STING, driving its translocation from the endoplasmic reticulum to the Golgi apparatus. STING recruits *TBK1*, leading to phosphorylation of *IRF3* and *NF-κB p65*. Phosphorylated *IRF3* dimers translocate to the nucleus and trigger robust transcription and secretion of type I interferons (IFN-α/β) and *CXCL9/10*. IFN-α/β paracrinally up-regulates MHC-I on tumor cells, enhances dendritic-cell (DC) maturation and cross-presentation, diminishes regulatory T cells (Tregs) and myeloid-derived suppressor cells (MDSCs) populations, and—via the *CXCL10–CXCR6* axis—recruits CXCR6^+^ CD8^+^ effector T cells into the tumor bed. Simultaneously, IR triggers immunogenic cell death (ICD) (95, 96). Additionally, radiotherapy causes DNA damage and IFN-γ (released by early-activated cytotoxic T lymphocytes (CTLs)) upregulates PD-L1 through the *JAK-STAT1* axis. Administration of PD-1/PD-L1 inhibitors at this juncture releases radiation-induced T-cell exhaustion. Recent studies show that low-dose radiotherapy (LDRT) combined with PD-1 inhibitors can induce stem-like CD8^+^ T cells from tumor-draining lymph nodes (TDLN) to the tumor and differentiate into CXCR6^+^ effector subpopulations, generating an abscopal effect, and forming long-term immune surveillance (97, 98).

LDRT reprograms the tumor microenvironment toward an immunostimulatory state with reduced immunosuppression and lower radiation toxicity compared with conventional radiotherapy, offering a rational strategy to sensitize immune−cold ES−SCLC to immunotherapy (99, 100). This synergistic potential is now corroborated by emerging preclinical and clinical evidence (101–103). Preclinical study and the phase II MATCH trial (NCT04622228) evaluated LDRT combined with PD-L1 inhibitors in ES- SCLC, well tolerated and produced durable responses: the confirmed overall response rate was 87.5% (95% CI 75.9–94.8%), median PFS 6.9 months (95% CI 5.4–9.3), and median OS 16.9 months (95% CI 14.0–32.9) (100). And this study also found that LDRT mobilized a quiescent, stem-like TCF1^+^PD-1^+^CD8^+^ T-cell subset within the tumor immune microenvironment in the murine models. Three-year follow-up confirmed sustained benefit, with PFS rates of 27.3% and 20.7% at one and three years, and OS rates of 69.6% and 35.1%, respectively (104). These data support further randomized evaluation of frontline LDRT plus chemoimmunotherapy for ES-SCLC.

Moreover, boron neutron capture therapy (BNCT) represents a precision radiotherapeutic modality that leverages boron-10 (^10^B)-labeled agents for selective tumor accumulation, generating short-range alpha particles upon neutron irradiation to achieve molecularly targeted destruction (105–107). Its distinct advantage lies in the dual capacity for direct tumor ablation coupled with immunostimulatory effects: tumor cell lysis releases HMGB1 and tumor-associated antigens that elicit systemic CD8^+^ T-cell responses, thereby inducing an abscopal effect (108). The first study combined BNCT with immunoprevention therapy to treat advanced brain tumors in rats in 2000 (105). BNCT was clinically approved in 2020 and exhibits remarkable tumor rejection in preclinical and clinical studies (109). Recently, BNCT has been integrated with immunotherapy as “boron neutron immunotherapy (B-NIT)”, which first demonstrated the ability to overcome immunotherapy resistance in malignant melanoma while preserving normal tissues through intratumoral dose confinement (110–112). B-NIT shows theoretical promise in SCLC, with advanced conjugates already developed—including neutron-triggered boron capsules, boron-rich polyboronate-ester micelles, and PD-L1 siRNA–loaded boron nanoparticles designed to elicit systemic antitumor immunity (108, 109, 113). However, clinical translation remains severely constrained by the extreme scarcity of neutron sources, currently limited to only a handful of specialized facilities. This infrastructure bottleneck necessitates prospective clinical validation before B-NIT can be meaningfully applied to SCLC.

In addition, combining radiotherapy with targeted small-molecule agents may enhance anti-PD-1 responses in SCLC, fostering systemic antitumor immunity and warranting further clinical exploration. Poly (ADP) ribose polymerase (PARP) plays a key role in DNA repair and is highly expressed in SCLC (114). The family of PARP enzymes are highly abundant nuclear proteins that mediate base excision repair (BER) and homologous recombination repair (HRR), and alternative end joining (a-EJ) (115). PARP1 detects and fixes DNA single-strand breaks (SSBs) by adding ADP-ribose to nearby proteins. PARP1 inhibitors trap the enzyme on the SSB; without NAD^+^ it cannot finish the repair, the replication fork stalls, the SSB becomes a double-strand break, and the cell dies by apoptosis (116, 117). PARP inhibition (PARPi) exhibits a strong radiosensitizing effect in SCLC cell lines and xenograft models (118, 119). In particular, talazoparib exhibited greater PARP trapping activity that was associated with superior radiosensitization (120). The PARPi combined with radiotherapy (PARPi/RT) activates the cGAS–STING pathway, up-regulating *CCL5* and *CXCL10* transcription, and—via *EIF4E2*-mediated stabilization of *CXCL10* mRNA—elevates *CXCL10* protein levels. PARPi also upregulated the protein and surface expression of PD-L1 and potentiated the cytotoxic effects of PD-L1 inhibitors in SCLC models (88). Therefore, addition of immunotherapy to PARPi/RT further augments tumor regression by enhancing T-cell infiltration and function. Zhang et al. showed that PARPi niraparib plus radiotherapy sensitizes tumors to immunotherapy, driving dense infiltration of cytotoxic and memory-effector T cells in preclinical SCLC models (121). Recently, Ran et al. reported that olaparib or talazoparib combined with radiotherapy and PD-L1 inhibitor significantly inhibited tumor growth in the B6129F mice bearing *KP1* tumors (119). Further flow cytometric analysis of the tumor microenvironment after treatment showed that the total infiltration of T cells into the tumors in the combined treatment group was significantly increased. Furthermore, combining radiotherapy and immunotherapy with other targeted small-molecule agents—such as STING agonists diABZI and the CDK4/6 inhibitor abemaciclib—may enhance anti-PD-1 responses in SCLC, fostering systemic antitumor immunity and warranting further clinical exploration (122, 123).

Radiotherapy ignites the cytosolic DNA–cGAS–STING–type I IFN circuit in SCLC, thereby transforming an immunologically “cold” tumor into an inflamed microenvironment rich in neoantigens and CXCR6^+^ CD8^+^ T cells (124). Concomitant PD-1/PD-L1 blockade then releases the adaptive PD-L1-mediated brake imposed by radiation, establishing a feed-forward loop in which radiotherapy opens a therapeutic window and immune-checkpoint inhibition secures it. This synergy systematically amplifies antitumor immunity and translates into durable survival benefit for ES-SCLC (**Table 2**). However, the optimal patient subset, radiation dose, timing, and neurotoxicity-mitigation strategies for chemoradiation with ICIs in ES-SCLC remain undefined, and prospective validation is urgently required. Moreover, delivering consolidative TRT during active immunotherapy may heighten the risk of immune-related pneumonitis, underscoring the need for precise patient selection, adaptive dosing schedules, and robust toxicity-monitoring protocols before this combination can be adopted as standard care.

**Table 2:** The current clinical trial stage of radiotherapy combined with immunotherapy in solid tumors or lung cancer.

Clinical trials.gov ID	Intervention	Clinical Trial Registration URL	Phase	Treatment line	Cancer stage	Status
Sequential radiotherapy VS. concurrent radiotherapy						
NCT06768307	/	https://clinicaltrials.gov/ct2/show/NCT06768307	II	First-line treatment	ES-SCLC	Not yet recruiting
NCT03223155	/	https://clinicaltrials.gov/ct2/show/NCT03223155	I	/	Metastatic Lung cancer	Active, not recruiting
Sequential thoracic radiotherapy						
NCT06586697	45 Gy/30 F	https://clinicaltrials.gov/ct2/show/NCT06586697	II	First-line treatment	ES-SCLC	Recruiting
NCT06125041	2Gy*(20-30) F	https://clinicaltrials.gov/ct2/show/NCT06125041	II	Maintenance treatment	ES-SCLC	Recruiting
NCT05617963	45 Gy/30 F	https://clinicaltrials.gov/ct2/show/NCT05617963	II	Maintenance treatment	LS-SCLC	Recruiting
NCT05557552	50Gy/25F	https://clinicaltrials.gov/ct2/show/NCT05557552	/	/	NSCLC	Recruiting
NCT06514118	50Gy/25F	https://clinicaltrials.gov/ct2/show/NCT06514118	II	Maintenance treatment	ES-SCLC	Recruiting
Concurrent thoracic radiotherapy						
NCT05552846	45 Gy/15 F	https://clinicaltrials.gov/ct2/show/NCT05552846	I	Maintenance treatment	ES-SCLC	Recruiting
NCT04624204	45 Gy/30 F	https://clinicaltrials.gov/ct2/show/NCT04624204	III	First-line treatment	LS-SCLC	Active, not recruiting
NCT02434081	66 Gy/33 F	https://clinicaltrials.gov/ct2/show/NCT02434081	II	First-line treatment	NSCLC	Completed
NCT03774732	18 Gy/6 F	https://clinicaltrials.gov/ct2/show/NCT03774732	III	First-line treatment	NSCLC	Active, not recruiting
NCT03275597	30–50 Gy/5 F	https://clinicaltrials.gov/ct2/show/NCT03275597	I	/	NSCLC	
NCT04765709	< 20 Gy	https://clinicaltrials.gov/ct2/show/NCT04765709	II	Maintenance treatment	NSCLC	Active, not recruiting
NCT03313804	30 Gy/10 F	https://clinicaltrials.gov/ct2/show/NCT03313804	II	Post-treatment	Advanced solid tumors	Active, not recruiting
Super-hyper fractionation pulse radiotherapy						
NCT05754203	8Gy/0.5Gy*16F	https://clinicaltrials.gov/ct2/show/NCT05754203	/	/	NSCLC	Recruiting
Reduced-dose hypo-fractionated thoracic radiotherapy						
NCT05128630	/	https://clinicaltrials.gov/ct2/show/NCT05128630	II	First-line treatment	NSCLC	Recruiting

### 4.3 ICIs + anti-angiogenic drugs

SCLC tumors are highly vascular and VEGF-rich, driving rapid progression (125–127). VEGF blockade normalizes chaotic tumor vasculature, lowers hypoxia (HIF-1α), increases CD8^+^ T cell infiltration, and reduces Tregs and MDSCs trafficking. Anti-angiogenic drugs now show promise when combined with chemoimmunotherapy, extending survival in early trials (128, 129). This review will summarize the latest anti-angiogenic drugs for the treatment of ES-SCLC.

Anlotinib received approval from the NMPA of China for third-line or subsequent treatment of ES-SCLC based on the ALTER 1202 trial (130). However, anlotinib given concurrently with PD-1/PD-L1 inhibitors as first or second-line maintenance therapy for ES-SCLC demonstrated encouraging efficacy and an acceptable safety profile: median PFS was 8.2 months, OS 20.1 months, and the ORR reached 50.0% in a single-center retrospective study (131). Encouragingly, Cheng et al. reported that first-line benmelstobart (anti-PD-L1) combined with anlotinib and etoposide/carboplatin (Anl/Ben/CT) significantly prolonged median OS compared with chemotherapy alone (19.3 vs 11.9 months; HR = 0.61; P = 0.0002), highlighting the potential of anti-angiogenic plus immunotherapy combinations in ES-SCLC in the phase III ETER701 trial (27, 132). A recent meta-analysis of 12 randomized controlled trials evaluating 15 first-line immunotherapy regimens for ES-SCLC corroborates the prognostic benefit observed with the Anl/Ben/CT triplet in the ETER701 study. The pooled analysis demonstrated that the Anl/Ben/CT regimen significantly reduced the risk of death compared with chemotherapy alone (HR 0.61, 95% CI 0.47–0.80). Bayesian ranking probabilities positioned the Anl/Ben/CT regimen first for both PFS (98.9%) and OS (41.4%) among the 15 evaluated regimens, and it also achieved the highest rank probability for overall response rate (ORR; 23.5%) (133). In addition to this, multiple meta-analyses indicated that chemoimmunotherapy combined with anti-angiogenesis agents represent a promising new therapeutic paradigm for ES-SCLC (134, 135). Even a subgroup analysis revealed patients under the age of 65 receiving anti-angiogenesis agents will achieve better survival outcomes (135).

Other anti-angiogenic drugs such as bevacizumab, when used in combination with etoposide and cisplatin, show promising application prospects in the treatment of ES-SCLC, improving PFS but not OS, and the frequency of ≥3 grade treatment-related adverse events (TRAEs) is higher (134, 136). In the phase II CeLEBrATE trial, the combination regimen of bevacizumab, atezolizumab, carboplatin/etoposide demonstrated encouraging first-line activity in ES-SCLC (137). The 1-year OS rate was 61.8% (90% CI 0.51–0.73; p = 0.040), with a median OS of 12.9 months (95% CI 11.6–17.5). Median PFS reached 6.2 months (95% CI 5.4–6.6), and the ORR was 83.3% (95% CI 69.8–92.5%). These data provide preliminary evidence supporting the integration of anti-angiogenesis with chemoimmunotherapy in ES-SCLC, warranting phase III validation. What’ s more, apatinib, a VEGFR2-targeting tyrosine kinase inhibitor, has also demonstrated promising anti-tumor activity in the combined treatment of SCLC (138). The PASSION trial demonstrated that camrelizumab plus apatinib confers promising antitumor activity and acceptable toxicity in second-line ES-SCLC, regardless of prior chemotherapy sensitivity (139). Recently, a multicenter, single-arm phase II study (NCT05001412) further indicated that this regimen yields superior survival outcomes and robust antitumor efficacy, supporting its potential as a first-line option for ES-SCLC (140). Ivonescimab is a humanized IgG1 bispecific anti-programmed cell death protein 1/vascular endothelial growth factor antibody. In a multicenter, open-label phase Ib study (NCT05116007), ivonescimab combined with chemotherapy was well tolerated and clinically active (among 35 enrolled patients, the confirmed ORR was 80% and the DCR 91.4%), supporting its evaluation in larger, controlled trials (141). However, apart from anlotinib, no other anti-angiogenic drugs have been officially approved by any national drug regulatory agency for this indication.

### 4.4 ICIs combined with small molecule targeted therapy

#### 4.4.1 DLL-3 targeted therapy

Delta-like ligand 3 (DLL3) is an emerging therapeutic target for SCLC. DLL3 is an inhibitory Notch ligand that is overexpressed in 70-80% of SCLC tumors but minimally expressed in normal tissues (142, 143). DLL3 is an atypical ligand for Notch receptor that lacks the N-terminal domain required for canonical Notch activation (144). Instead, DLL3 binds Notch in cis within the Golgi-endosomal compartment, forming an intracellular DLL3–Notch complex that prevents receptor maturation and surface localization. This cis-inhibition blocks binding of canonical ligands such as *DLL1/4*, thereby suppressing Notch intracellular domain (NICD) release and down-regulating *HES1/HEY1 (*145). In addition, DLL3 is regulated by *ASCL1*, a transcription factor prevalent in the SCLC-A subtype (146). The resulting *HES1* low, *ASCL1* high transcriptional program locks cells in an undifferentiated neuroendocrine state, preserving stem-like properties and driving continuous proliferation (144). Independently of Notch signaling, DLL3 upregulates the transcription factor *SNAI1* in SCLC, triggering epithelial-to-mesenchymal transition (EMT) (98, 142). This leads to E-cadherin loss, N-cadherin and vimentin up-regulation, and significantly enhances tumor-cell migration and invasion. The SCLC-A subtype has a high expression of *ASCL1*, so its level of *DLL3* is significantly higher than that of other subtypes, accounting for approximately 50-60% of all SCLC, providing an enriched “target population” for DLL3-targeted therapy. Currently, the anti-tumor drugs targeting the *DLL3* in SCLC mainly include antibody-drug conjugates (ADCs), bispecific T-cell engagers (BiTEs), and CAR-T cell therapy. Xenograft data revealed PD-1 blockade significantly augmented BiTEs efficacy (147).

Rovalpituzumab tesirine (Rova-T) is the first DLL3-targeted ADC. It contains a humanized specific IgG1 monoclonal antibody targeting DLL3, a pyridopyridoxine dithiocarbamate cytotoxin, and a cleavage linker (148). Phase I data showed a 38% ORR in patients with ≥50% DLL3-expressing tumor cells, and the Phase II TRINITY study further confirmed Rova-T’s efficacy across SCLC patients with varying DLL3 levels (149, 150). However, two subsequent Phase III trials—one evaluating maintenance therapy after first-line platinum-based chemotherapy and another comparing Rova-T with topotecan as second-line therapy—were discontinued because of limited efficacy and toxicity concerns (151, 152). DB-1314, a novel DLL3-targeting ADC with DNA topoisomerase I inhibitor, exhibits promising safety profile and therapeutic efficacy in preclinical SCLC models (153). In addition, FZ-AD005, a next-generation DLL3-directed ADC, combines the humanized antibody FZ-A038 with a Val-Ala cleavable linker–payload DXd (154).In Cell line-derived xenograft (CDX) and patient-derived xenografts (PDX) models it achieved robust, dose-dependent tumor regressions; cynomolgus PK showed high stability and acceptable exposure. Repeat-dose toxicology in rats and monkeys revealed no notable toxicities, indicating a favorable safety margin (154). These data support FZ-AD005 as a promising, well-tolerated DLL3 ADC for SCLC therapy. The combination of ADCs with PD-1/PD-L1 inhibitors is poised to become a new treatment paradigm for SCLC, with the DLL3-targeted ADC ZL-1310 now in clinical trials alongside atezolizumab (NCT06179069).

Tarlatamab (AMG 757) is the first-in-class DLL3-targeted bispecific T-cell engager (BiTE). It consists of two single-chain variable fragments (scFvs)—one that binds DLL3 on tumor cells and another that engages CD3 on T cells—fused to an Fc region that extends serum half-life. By simultaneously tethering DLL3-positive cancer cells and CD3-positive T cells, Tarlatamab drives MHC-unrestricted T-cell activation, prompting release of granzyme B and perforin and inducing rapid tumor-cell lysis (148). Tarlatamab has shown superior survival and a manageable safety profile in a pivotal phase III trial, positioning it to redefine second-line therapy for SCLC. Moreover, phase I data from DAREON^®^-9, presented at ASCO 2025, show obrixtamig (BI 764532) is promising for SCLC. In addition, trispecific T-cell engagers (TiTEs), HPN328 (MK-6070), its phase I/II trial (NCT04471727) assesses single-agent or combination atezolizumab/ifinatamab-deruxtecan in DLL3-positive high-grade neuroendocrine tumors, including SCLC. Multiple clinical trials combining BiTEs with other immunotherapies are now being explored in ongoing studies(**Table 3**).

**Table 3:** Clinical trials of targeted therapy combined immunotherapy in solid tumors and lung cancer.

Clinical trials.gov ID		Drug	Clinical Trial Registration URL	Phase	Cancer stage	Status
DLL-3 targeted treatment						
ADC	NCT06179069	ZL-1310	https://clinicaltrials.gov/ct2/show/NCT06179069	I	ES-SCLC	Recruiting
BiTEs	NCT05361395	Tarlatamab	https://clinicaltrials.gov/ct2/show/NCT05361395	I	ES-SCLC	Active, not recruiting
	NCT06211036	Tarlatamab	https://clinicaltrials.gov/ct2/show/NCT06211036	I	ES-SCLC	Recruiting
	NCT04885998	Tarlatamab	https://clinicaltrials.gov/ct2/show/NCT04885998	III	SCLC	Completed
	NCT06898957	Tarlatamab	https://clinicaltrials.gov/ct2/show/NCT06898957	I	ES-SCLC	Recruiting
	NCT05879978	Obrixtamig(BI 764532)	https://clinicaltrials.gov/ct2/show/NCT05879978	I	SCLC	Active, not recruiting
	NCT06077500	Obrixtamig(BI 764532)	https://clinicaltrials.gov/ct2/show/NCT06077500	I	SCLC	Recruiting
TiTE	NCT04471727	HPN328	https://clinicaltrials.gov/ct2/show/NCT04471727	I/II	Advanced Cancers	Recruiting
PARP inhibitors						
	NCT02734004	Olaparib	https://clinicaltrials.gov/ct2/show/NCT02734004	II	LS-SCLC	Active, not recruiting
	NCT04538378	Olaparib	https://clinicaltrials.gov/ct2/show/NCT04538378	II	EGFR-Mutated LUAD transform to SCLC	Terminated
	NCT04728230	Olaparib	https://clinicaltrials.gov/ct2/show/NCT04728230	I	NSCLC	Active, not recruiting
	NCT02484404	Olaparib	https://clinicaltrials.gov/ct2/show/NCT02484404	II	NSCLC	Unknown
	NCT02660034	Pamiparib	https://clinicaltrials.gov/ct2/show/NCT02660034	I	Advanced solid tumors	Active, not recruiting
	NCT04701307	Niraparib	https://clinicaltrials.gov/ct2/show/NCT04701307	II	SCLC	Active, not recruiting
	NCT04334941	Talazoparib	https://clinicaltrials.gov/ct2/show/NCT04334941	II	SLFN11 Positive SCLC	Active, not recruiting
	NCT03958045	Rucaparib	https://clinicaltrials.gov/ct2/show/NCT03958045	II	SCLC	Completed

ADC, antibody-drug conjugates; BiTEs, bispecific T-cell engagers; TiTE, trispecific T-cell engager.

Lastly, AMG 119, a DLL3-targeting CAR-T cell therapy, has demonstrated manageable safety and preliminary efficacy signals in patients with DLL3-expressing relapsed/refractory SCLC in a phase I trial (NCT03392064) (155). Combination strategies—including co-administration with ICIs—are under investigation to mitigate T-cell exhaustion and enhance antitumor activity. In conclusion, the combined strategy of DLL3-targeted therapy and ICIs is expected to overcome the immune escape characteristics of SCLC and improve patient prognosis.

#### 4.4.2 Aurora A kinase inhibitors

A small population of SCLC extinguishes the *ASCL1*-driven neuroendocrine program while re-engaging innate-immune signaling. These “inflammatory” SCLC sustain durable remissions under PD-1/PD-L1 blockade. Aurora A kinase (AURKA) is recurrently overexpressed in SCLC and orchestrates centrosome maturation and spindle assembly (156). Some SCLC are highly sensitive to Aurora kinase inhibitors. The Aurora A kinase inhibitor (AURKAi) LSN3321213 combined with the PD-L1 inhibitors, achieved persistent anti-tumor efficacy in the immunocompetent SCLC genetically engineered mouse models (GEMMs) and syngeneic xenografts: LSN3321213 arrested tumor cells in the mitotic phase (M phase), restored interferon signal transduction, increased the sensitivity of tumor cells to PD-L1 inhibitor; simultaneously, it induced high interferon signaling and MHC-I, promoting CD8^+^ T cell-mediated tumor cell killing (157). The combination of AURKA inhibitor and PD-L1 further expanded intratumoral CD8^+^ effector and CD4^+^ memory T-cell infiltrates, amplifying anti-tumor immunity. Importantly, AURKAi spared lymphocyte proliferation, providing a therapeutic window that selectively targets cancer cells while preserving immune competence. Further clinical trials are needed to verify this result.

#### 4.4.3 PARP inhibitors

SCLC cell lines and tumors exhibited an elevated level of PARP 1 protein and mRNA compared to healthy lung tissues and other subtypes of lung tumors, especially the SCLC-P subtype, which is defined by the significant expression of the transcription factor *POU2F3* (114). Previous preclinical investigation showed that PARPi potentiates chemotherapy and radiation *in vitro* and *in vivo* in SCLC. PARPi combined with chemotherapy significantly inhibited the growth of SCLC tumors in preclinical models, but no significant benefits were observed in the related clinical trials (118, 158, 159). An increasing number of studies have shown that the DNA damage response is associated with anti-tumor immunity in various cancers (including SCLC), providing a theoretical basis for combining PARPi and immunotherapy regimens to achieve a synergistic effect (160–162). The combination of olaparib and durvalumab exerts antitumor activity, yielding modest efficacy (ORR 10–15%; mPFS 1.8–2 months) with acceptable tolerability in relapsed SCLC(NCT02734004、NCT02484404) (163, 164). Furthermore, pamiparib combined with tisotumab vedotin demonstrated varying degrees of anti-tumor activity in patients with advanced solid tumors (NCT02660034) (165, 166). In the future, further studies will be conducted to investigate the efficacy of the combined treatment regimen of pamiparib and tislelizumab in SCLC. Recently, talazoparib is a new generation of PARPi and is gradually entering the clinical exploration stage for combined immunotherapy in SCLC. It is particularly suitable for patients with biomarker screening (such as high expression of *SLFN11*). Karim et al. reported that maintenance atezolizumab plus talazoparib prolonged PFS in patients with *SLFN11*-positive ES-SCLC but was associated with increased hematologic toxicity, primarily grade 3 anemia (29). Several similar clinical trials (NCT04701307, NCT04334941, NCT04538378, and NCT03958045) are currently recruiting SCLC patients to evaluate the use of PARPi and anti-PD1 antibody combination therapy (**Table 3**). However, clinical evidence for PARPi combined with ICIs in SCLC remains scarce. Most evidence comes from small, non-randomized studies; adequately powered, comparative, and double-blind trials are still needed to validate the benefit of this combination.

### 4.5 Epigenetic regulation drugs

Epigenetic disruption is now recognized as a central driver of tumorigenesis. Recently, several pre-clinical and early-phase studies have combined histone deacetylase inhibitors (HDACi) (e.g., vorinostat, entinostat) or DNA methyltransferase inhibitors (DNMTi) (e.g., azacitidine, decitabine) with ICIs in NSCLC (167–171). These regimens aim to remodel the tumor microenvironment—enhancing antigen presentation, elevating T-cell infiltration, and up-regulating checkpoint ligands—thereby augmenting the response to PD-1/PD-L1 or CTLA-4 inhibitors.

Epigenetic mechanisms may regulate the distinction between SCLC-A and SCLC-N models (35). Mohammad et al. establish the histone demethylase Lysine Demethylase 1 (LSD1) as a tractable therapeutic vulnerability in SCLC (172, 173). LSD1 is a histone modifier that sustains embryonic stem cell pluripotency by removing methyl marks from histone H3 lysine 4 (H3K4), thereby silencing genes that would otherwise drive differentiation (174). The LSD1 inhibitor T-3775440 suppresses SCLC proliferation by disrupting the interaction between LSD1 and the SNAG-domain proteins insulinoma-associated protein 1 (INSM1) and growth-factor-independent 1B (GFI1B) (175). The downstream consequences of INSM1 repression are largely mediated by *ASCL1*—a master regulator of neuroendocrine differentiation that reshapes INSM1-dependent neuroendocrine transcriptional programs in SCLC cells. In the both human SCLC cell lines and immunocompetent mouse models, LSD1 inhibition restored surface MHC-I, transcriptionally activated antigen-presentation genes, and engaged interferon signaling, rendering SCLC cells susceptible to MHC-I-restricted T cell cytolysis (176). These findings position LSD1 as a key gatekeeper of MHC-I antigen presentation, offering a mechanistic basis for pairing LSD1 blockade with immune-checkpoint inhibitors to enhance outcomes in SCLC. Moreover, Hiatt et al. showed that, in an *Rb1/Tp53*-deficient, syngeneic and immunocompetent SCLC model, co-treatment with the LSD1 inhibitor bomedemstat and PD-1 blockade markedly expanded intratumoral CD8^+^ T cells and produced robust tumor growth inhibition—findings that now underpin a planned clinical trial combining bomedemstat with standard PD-1 axis therapy in SCLC (177). In short, epigenetic therapies appear to be a promising novel therapy for SCLC, offering an incremental step toward more patient-tailored approaches.

## 5 Discussion and perspectives

Landmark phase III trials (IMpower133, CASPIAN, ASTRUM-005) have established PD-1/PD-L1 inhibitors plus etoposide–platinum chemotherapy as the first-line standard for ES-SCLC, extending median OS from 8–10 months with chemotherapy to 12–15 months and 2-year OS rate to 20–25%. Despite these gains, ES-SCLC remains clinically challenging. Primary resistance occurs in ~60% of patients, robust predictive biomarkers are lacking (PD-L1 is rarely expressed and not predictive; tumor mutational burden has limited utility), cumulative immune-related and cytotoxic toxicities complicate management, and high costs restrict global access.

Next-generation strategies are now under intensive investigation. Cellular immunotherapy (ex vivo-expanded natural killer cells plus atezolizumab); the radioligand 177Lu-DOTATATE (Lutathera) combined with nivolumab have shown acceptable safety and early efficacy signals (178, 179). Oncolytic viruses convert the “cold” SCLC microenvironment into a T-cell-inflamed phenotype, sensitizing tumors to PD-1/PD-L1 blockade (180, 181). Small-molecule DNA-damage–response inhibitors potentiate checkpoint blockade through distinct but complementary mechanisms. Ataxia telangiectasia and rad3 related inhibitors trigger STING-dependent interferon signaling and up-regulate *MHC-I*, sensitizing SCLC to PD-L1 blockade in pre-clinical models and patient specimens (182). Similarly, PARP or CHK1 inhibition increases tumor-cell PD-L1 expression, triggering pronounced CD8^+^ T-cell infiltration and robust antitumor activity; CD8^+^ T-cell depletion completely abrogates this synergy, confirming their essential role in the combined DDR inhibitor/PD-L1 strategy (88). PFKFB4-directed biomimetic co-delivery system induces ferroptosis while simultaneously enhancing anti-PD-L1 activity (183). In addition, dual checkpoint suppression is being tested with serplulimab (anti-PD-1) plus TIGIT or LAG-3 inhibitors, a regimen that depletes intratumoral regulatory T cells, expands effector and memory CD8+ T cell pools, and broadly reprograms immune-related gene expression (184, 185), while the Toll-like receptor 9 (TLR9) agonist lefitolimod reactivates innate and adaptive immune surveillance to eliminate minimal residual disease (186).

Despite these encouraging developments, several challenges must be addressed before such strategies can be broadly implemented. The remarkable heterogeneity and rapid adaptability of ES-SCLC suggest that future breakthroughs will hinge on our ability to understand and target each tumor’s unique molecular ecosystem. Integrated multi-omics analysis is the key to achieving this. By moving beyond single-layer genomic or transcriptomic perspectives, we can deconvolute the intricate interplay between tumor cells and the immune microenvironment. Future efforts should focus on prospectively validating molecular and immune microenvironment-based biomarkers, developing rational combination strategies that incorporate metabolic or epigenetic modulators, and advancing next-generation immunotherapeutics such as bispecific antibodies and neoantigen-based cancer vaccines. Through the integration of precision stratification with innovative immunotherapy platforms, ES-SCLC may transition from an exceptionally recalcitrant disease to one amenable to durable, individualized control.

## Glossary

SCLC: Small-cell lung cancer
NSCLC: non-small cell lung cancer
ES-SCLC: Extensive-stage small-cell lung cancer
LS-SCLC: Limited-stage small-cell lung cancer
ASCL1: achaete-scute homologue 1
NeuroD1: neurogenic differentiation factor 1
POU2F3: POU class 2 homeobox 3
YAP1: yes-associated protein 1
NE: neuroendocrine
VALG: Veterans Administration Lung Study Group
FDA: Food and Drug Administration
EMA: European Medicines Agency
NMPA: The National Medical Products Administration
CSCO: The Chinese Society of Clinical Oncology
RT: radiotherapy
LDRT: low-dose radiotherapy
BiTEs: DLL3-directed bispecific T-cell engagers
PARPi: PARP inhibition
HDACi: histone deacetylase inhibitors
DNMTi: DNA methyltransferase inhibitors
ICIs: immune checkpoint inhibitors
PFS: progression free survival
OS: overall survival
ORR: objective response rate
HR: hazard ratio
NMF: non-negative matrix factorization
EMT: epithelial-mesenchymal transition
CTLA-4: cytotoxic T lymphocyte-associated antigen 4
PD-1: programmed cell death protein 1
PD-L1: programmed death ligand 1
PD-L2: programmed death ligand 2
TCR: T cell receptor
ITIM: immunoreceptor tyrosine-based inhibitory motif
ITSM: immunoreceptor tyrosine-based switch motif
SHP-2: src homology 2 domain-containing tyrosine phosphatase 2
ZAP70: zeta-chain-associated protein kinase 70
SLP-76: src-like adapter protein of 76 kda
PKC-θ: protein kinase C θ
PI3K: phosphoinositide-3-kinase
N-SH2: N-terminal src homology 2 domain
C-SH2: C-terminal src homology 2 domain
APCs: antigen-presenting cells
NKs: natural killer cells
DC: dendritic-cell
Tregs: regulatory T cells
MDSCs: myeloid-derived suppressor cells
CTLs: cytotoxic T lymphocytes
TDLN: tumor-draining lymph nodes
TAAs: tumor-associated antigens
TRAEs: treatment-related adverse events
PARP: Poly (ADP) ribose polymerase
IR: Ionizing radiation
ROS: Reactive oxygen species
DSBs: DNA double-strand breaks
cGAS: cyclic GMP–AMP synthase
ICD: immunogenic cell death
BER: base excision repair
HRR: homologous recombination repair
a-EJ: alternative end joining
SSBs: single-strand breaks
NICD: Notch intracellular domain
EMT: epithelial-to-mesenchymal transition
ADCs: antibody-drug conjugates
BiTEs: bispecific T-cell engagers
Rova-T: Rovalpituzumab tesirine
CDX: Cell line-derived xenograft
PDX: patient-derived xenografts
AURKA: Aurora A kinase
AURKAi: Aurora A kinase inhibitor
GEMMs: genetically engineered mouse models
M phase: mitotic phase
LSD1: Lysine Demethylase 1
H3K4: histone H3 lysine 4
INSM1: insulinoma-associated protein 1
GFI1B: growth-factor-independent 1B
TLR9: Toll-like receptor 9
